# Dr. Bolton’s Cases of Foreign Bodies Removed from the Ear and Esophagus

**Published:** 1842-09-24

**Authors:** James Bolton

**Affiliations:** Richmond, Va.


### *Cases of Foreign Bodies removed from the Ear and Esophagus.*

By James Bolton, M. D.

(Communicated through Professor Dunglison.)

*Foreign body in the ear seven years.*—During the latter part of last January, I was called to examine a girl, about thirteen years old, who complained of pain and deafness, with occasionally annoying sounds in one ear. From the closest inquiry into the history of the case, I could learn nothing which gave the least explanation of these symptoms, except that, on reflection, her mother remembered being alarmed by the child coming to her, when she was abont six years old, and telling her that she had got a grain of coffee in her ear. Not having the aid of bright sunshine, I could not make a satisfactory examination, although I discovered a dark substance, which I supposed to be inspissated cerumen, lying apparently upon the tympanum. I therefore syringed the ear with warm water and castile soap. On throwing in about the third syringeful with some force, a black looking substance shot into the basin, which on examination proved beyond all doubt, to be half of a coffee grain. She has been ever since, entirely free from any unpleasant feeling in the ear, and has recovered her hearing.

It is stated in various Journals, that Carpenter has successfully employed cold water injection, for the removal of foreign bodies from the ear.

*Foreign body in the Esophagus(?) seventeen years.*—A highly intelligent young lady of this city, whose health has always been delicate, on rising in the morning about two years since, coughed slightly and hawked some hard substance into her mouth. On removing a small quantity of mucus, with which it was ejected, it was discovered to be a small green glass ear drop. Her mother recollected distinctly, that a pair of ear rings, with green glass drops, had been presented to her by a relative, seventeen years previously, and that shortly afterwards, she had complained of having swallowed one of them; but as she felt no inconvenience, no notice was taken of it. The intelligence and veracity of the lady are such, as place the facts as related beyond all doubt. The ear drop, too, is evidently a very old fashioned affair, such as is not now to be found in any jeweller's shop.

*Richmond, Va., August*, 1842.

